# Stacking Interactions between Carbohydrate and Protein Quantified by Combination of Theoretical and Experimental Methods

**DOI:** 10.1371/journal.pone.0046032

**Published:** 2012-10-08

**Authors:** Michaela Wimmerová, Stanislav Kozmon, Ivona Nečasová, Sushil Kumar Mishra, Jan Komárek, Jaroslav Koča

**Affiliations:** 1 CEITEC - Central European Institute of Technology, Masaryk University, Brno, Czech Republic; 2 National Centre for Biomolecular Research, Faculty of Science, Masaryk University, Brno, Czech Republic; Indian Institute of Science, India

## Abstract

Carbohydrate – receptor interactions are an integral part of biological events. They play an important role in many cellular processes, such as cell-cell adhesion, cell differentiation and in-cell signaling. Carbohydrates can interact with a receptor by using several types of intermolecular interactions. One of the most important is the interaction of a carbohydrate's apolar part with aromatic amino acid residues, known as dispersion interaction or CH/π interaction. In the study presented here, we attempted for the first time to quantify how the CH/π interaction contributes to a more general carbohydrate - protein interaction. We used a combined experimental approach, creating single and double point mutants with high level computational methods, and applied both to *Ralstonia solanacearum* (RSL) lectin complexes with α-l-Me-fucoside. Experimentally measured binding affinities were compared with computed carbohydrate-aromatic amino acid residue interaction energies. Experimental binding affinities for the RSL wild type, phenylalanine and alanine mutants were −8.5, −7.1 and −4.1 kcal.mol^−1^, respectively. These affinities agree with the computed dispersion interaction energy between carbohydrate and aromatic amino acid residues for RSL wild type and phenylalanine, with values −8.8, −7.9 kcal.mol^−1^, excluding the alanine mutant where the interaction energy was −0.9 kcal.mol^−1^. Molecular dynamics simulations show that discrepancy can be caused by creation of a new hydrogen bond between the α-l-Me-fucoside and RSL. Observed results suggest that in this and similar cases the carbohydrate-receptor interaction can be driven mainly by a dispersion interaction.

## Introduction

Carbohydrate - protein interactions are incorporated into a wide range of biologically relevant processes [1]. These interactions are co-responsible for such fundamental mechanisms as cell growth, cell differentiation, energy storage, cell adhesion and other important processes [2]. Additionally, carbohydrate interactions and recognition in biological systems are related to many diseases, such as diabetes, viral and bacterial infections [3], [4], lysosomal storage disorders [5], inflammatory processes [6], and immune system response [7], [8]. All of the above mentioned phenomena are co-mediated by interactions between carbohydrates and their specific protein-based receptors. These receptors utilize several types of interactions to bind the carbohydrate moiety [9], [10]. It is generally considered that mainly hydrogen bonds between carbohydrate hydroxyl groups and polar amino acid residues are of key importance. It is also known that protein – carbohydrate interactions are often mediated by metal ions, such as calcium, zinc or magnesium. The aforementioned ions form positive bridges between oxygen atoms of the carbohydrate hydroxyl group and negatively charged protein residues. Lectins are an illustrative example, as the carbohydrate - receptor interaction is mediated by Ca^2+^ ions. These types of interactions are well known and described. However, the mutual positions of carbohydrate apolar faces and aromatic amino acid residues found in crystallographic structures of protein - carbohydrate complexes quite often indicate another type of contact classified as a van der Waals or hydrophobic interaction. It was proven, in the last few years that this is a specific type of interaction where London dispersion forces [11], [12] are mainly contributing. Inspection of protein-carbohydrate complexes in the PDB database reveals that this interaction occurs in many different carbohydrate processing enzymes, ranging from glycosidases through to transglycosidases or glycosyltransferases to carbohydrate-recognizing proteins, including lectins, immunoglobulins, glycosaminoglycans and many others [13].

Recently, the importance of this type of dispersion-driven interaction in biologically essential protein-carbohydrate complexes has been discussed in the literature [14]–[17], and there are several recently published papers that prove the presence of this type of interaction [12], [18]–[22]. Dispersion interactions between carbohydrates and aromatic moieties were also detected by solution NMR [15], [23]–[28] and other experimental methods [29]–[31].

On the other hand, there is currently no clear and detailed description of the behavioral properties of dispersion interactions specific to apolar carbohydrate parts. Recently, calculated interaction energies of carbohydrate - aromatic moiety complexes were determined only for specific mutual orientations obtained from single experimental structures of carbohydrate-protein complexes [22], [32], [33] and for several model systems [12], [33]–[36]. The results of such studies, especially concerning the nature of the protein carbohydrate interaction, correspond well with computational studies by Hobza and coworkers on oligonucleotides [37], [38]. They have shown that the stacking interactions, which are based mainly on dispersion part, are essential and stabilize a typical helix structure in DNA or RNA double helixes. It has been shown recently that also carbohydrate molecules can make stacking interactions with DNA duplexes [39]. Taking into account the wide-ranging occurrence of dispersion interactions, it becomes clear that a detailed understanding of dispersion interactions between carbohydrates and aromatic residues in proteins is very important as it may have important consequences, for example in the more precise drug design of carbohydrate-mimicking molecules [40], [41], or for other bioanalytical and biotechnology applications.

**Figure 1:**
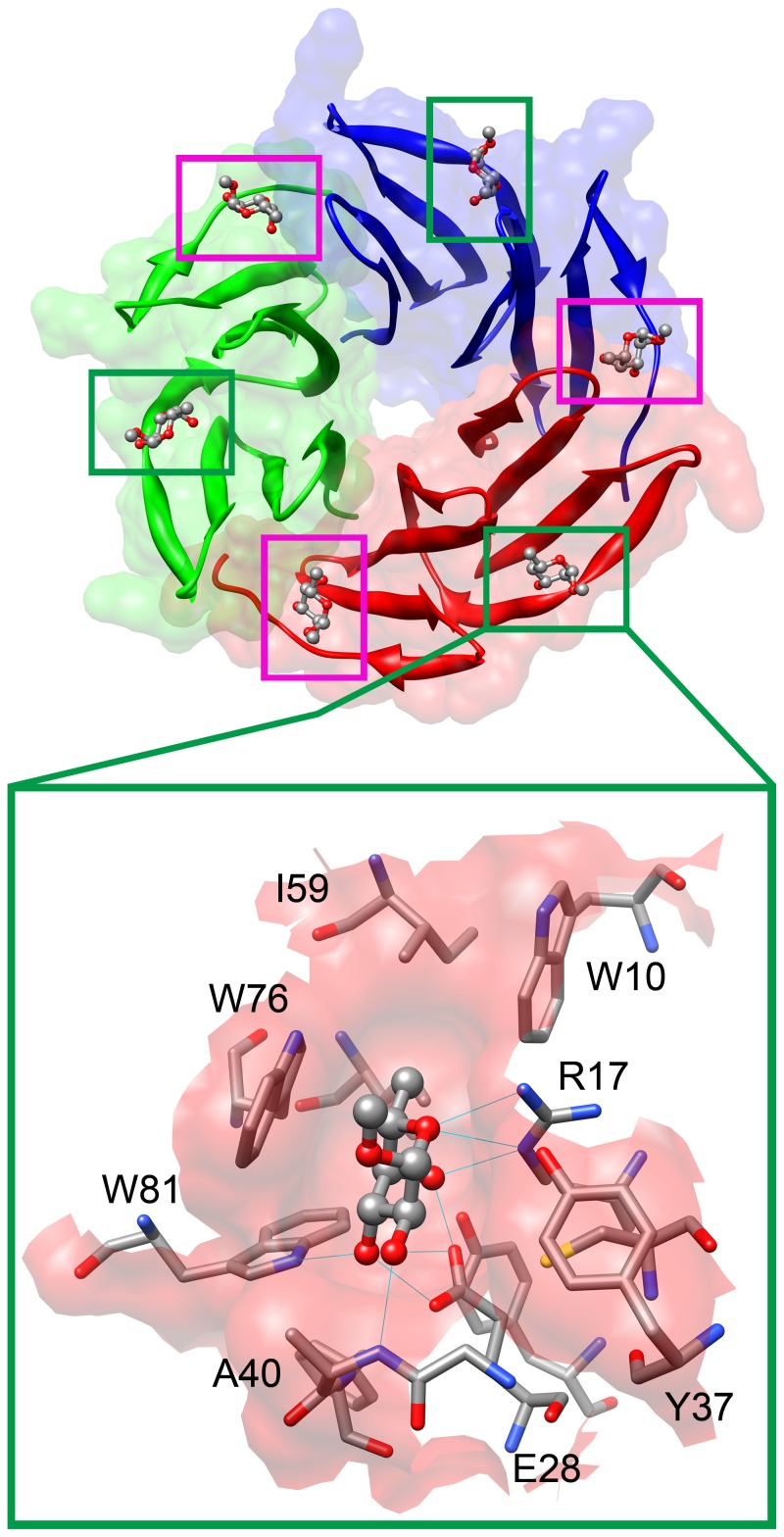
Visualization of the RSL lectin tertiary structure with binding site detail. The RSL lectin has six binding sites, marked by squares (top). Three intramonomeric (green squares) and three intermonomeric (magenta squares) binding sites are present in RSL. Detail of the intramonomeric binding site is shown on the bottom. The 2BT9 pdb crystal structure was used. For comparison of the binding sites see Supplementary Information (Figure S1).

For the purpose of this study, we chose fucose binding lectin RSL from *Ralstonia solanacearum* [42] as a model system (Figure 1). *Ralstonia solanacearum* is a Gram - negative β-proteobacterium, inhabiting water and soil and causing lethal wilt in more than 200 plants worldwide [43]. Found in the soil, it enters plant roots via wounds or secondary roots, invades the xylem vessels and subsequently spreads throughout the plant [44]. Each year, this bacterium causes major agronomic and economic losses in tropical climates. More recently, it threatens potato crops in temperate climates, due to the extension of strains adapted to cooler environmental conditions in Europe and North America. Therefore, RSL lectin is a suitable candidate to study the dispersion type interaction in carbohydrate – protein complexes. This lectin exhibits very high affinity to the α-l-fucose moiety (K_d_ for the α-l-Me-fucoside is 0.73 µM) [42] and the question arises as to why this is so. Comparable to RSL, such strong binding affinities to carbohydrates are only exhibited by lectins with metal ions included in the binding site, where strong polar interactions are involved in binding (for example PA-IIL lectin from *Pseudomonas aeruginosa* [45]). However, this type of interaction is not the case in this study, as there is no ion in the RSL active site, and rather binding is more likely due to a non-polar interaction of this lectin with α-l-fucose. The RSL crystal structure shows one tryptophan residue interacting with bound fucose through a stacking CH/π interaction in the binding site. In this study, we have attempted to quantify this interaction by means of site-directed mutagenesis combined with microcalorimetry as well as high level quantum chemical calculations complemented by molecular dynamics.

## Results and Discussion

### Experimental part of work

To obtain the most accurate experimental information about the contribution of CH/π interactions to the binding energy in protein/carbohydrate interactions, several RSL mutants were designed and prepared *in vitro*. As RSL contains two almost identical sets of intra and intermolecular binding sites (Figure 1 and Figure S1), we had to ensure that both of them were included in substitution experiments. Seven RSL mutants were prepared, consisting of four one-point mutants: W76F, W76A (in the intramolecular site), W31F, and W31A (in the intermolecular site); and three double-mutants: W31FW76F, W31AW76A and W31AW76F. Oligomeric state verification for all proteins was performed using size-exclusion chromatography (SEC) showing no significant change in their protein mobilities. However, RSL and its mutants display apparently lower molecular mass than expected, which we have already seen for several lectins with a compact structure containing only β-sheets. For example, the apparent molecular mass of carp egg lectin using the silica-based TSK G3000 SW resin is approximately half the value predicted for a single polypeptide chain. When using Superdex 75 resin, the protein moves as a monomer [46]. Similarly, Watanabe et al [47] showed that the apparent molecular weights of C-type lectins from eggs of shishamo smelt could not be estimated by SEC because of their nonspecific retention on various column matrices. Therefore, we also employed analytical ultracentrifugation as a more sophisticated method to determine their oligomeric form. The ultracentrifugation was done for the wild type RSL and W31A mutant only because the residue 31 is in the binding site on the edge between the monomers. The results confirmed that the proteins are trimers in a solution (Figure S2). As W31A mutant showed that the structure remains trimeric, it was not necessary do it for the other mutants as there is no reason why other mutations would affect oligomerization. This was also confirmed by SEC, where all proteins, wild type and all mutants, showed almost identical retention time.

Thermodynamics of RSL and its mutant's interactions with α-l-Me-fucoside, measured by isothermal titration calorimetry, is summarized in Table 1. As seen from the data, single-point substitutions of the stacking tryptophans by alanine (W31A, W76A) didn't change the affinity of the protein toward its ligand. However, mutations led to a drop of the ligand/RSL monomer stoichiometry to one. Based on these results, we prepared a double alanine mutant (W31AW76A). We were able to purify the protein by affinity chromatography, which was a signal of some residual ability to bind sugars amplified through the multivalency of the protein. ITC measurements showed that the affinity decreased by more than three orders of magnitude. Such low affinity is negligible compared to the affinity of the native protein. Using thermodynamic values obtained for W31AW76A alanine double-mutant, we recalculated binding data for both single-point alanine mutants (W31A and W76A) using two independent binding site fitting functions; nevertheless, the results were almost identical (for example, ΔG values for the W76A mutant calculated from three independent measurements were −8.38±0.038 and −8.41±0.039 kcal.mol^−1^ for one site and two sites models, respectively). During the fitting procedures, the stoichiometry parameter *n* has never been constrained. The obtained data, using the model for one independent site, has shown drop in apparent stoichiometry for single-point Ala mutants (W31A, W76A) to one, while for single point Phe mutants (W31F, W76F) has remained two.

**Table 1:** Thermodynamics of binding for wild type RSL and its mutants with α-l-Me-fucoside by ITC at 293 K (standard deviations were calculated from three independent measurements).

	*n*	K_A_×10^6^ (M^−1^)	K_D_×10^−6^ (M)	ΔG (kcal.mol^−1^)	ΔH (kcal.mol^−1^)	−TΔS (kcal.mol^−1^)	*E*_Int_ (kcal.mol^−1^)
**RSL**	**1.98 (±0.1)**	**1.6 (±0.20)**	**0.6 (±0.07)**	**−8.5(±0.07)**	**−11.1 (±0.01)**	**2.7 (±0.1)**	**−8.85**
W31F	1.97 (±0.1)	1.1 (±0.03)	0.9 (±0.02)	−8.3 (±0.01)	−9.9 (±0.13)	1.7 (±0.14)	n.d.
W76F	1.95 (±0.1)	1.30 (±0.02)	0.8 (±0.2)	−8.3 (±0.03)	−11.6 (±0.14)	3.3 (±0.1)	n.d.
**W31FW76F**	**2.14 (±0.1)**	**0.15 (±0.002)**	**6.9 (±0.02)**	**−7.1 (±0.002)**	**−9.0 (±0.04)**	**1.9 (±0.04)**	**−7.92**
W31AW76F	0.92 (±0.1)	0.08 (±0.001)	13.1 (±0.02)	−6.7 (±0.00)	−9.2 (±0.34)	2.6 (±0.3)	n.d.
**W31AW76F** [a]	**0.87 (±0.1)**	**0.12 (±0.13)**	**8.1 (±0.31)**	**−7.0 (±0.12)**	**−8.9 (±0.33)**	**1.9 (±0.30)**	n.d.
W31A	0.91(±0.1)	1.1(±0.03)	0.9 (±0.02)	−8.2 (±0.01)	−9.9 (±0.34)	1.7 (±0.10)	n.d.
W76A	0.99 (±0.0)	1.4 (±0.09)	0.7 (±0.05)	−8.4 (±0.04)	−11.2 (±0.13)	2.8 (±0.17)	n.d.
**W31AW76A**	**1.89 (±0.1)**	**0.0011(±2E^−5^)**	**926 (±32)**	**−4.1 (±0.23)**	**−7.8 (±0.21)**	**3.7 (±0.13)**	**−0.91**

The *E*_Int_ represents calculated interaction energy for a specific binding site model, *n* stands for stochiometry. Single point alanine mutations clearly show the stoichiometry change.

[a] fitted for two independent binding sites, parameters for W31A fixed.

To characterize affinity changes connected with the phenylalanine substitution, we have considered also several other mutants. Single-point mutants (W31F and W76F) showed a slight decrease in binding energies when fitted in the same manner as the wild-type protein, which indicated worse binding of the mutants compared to the native protein. As both binding sites have a similar architecture, the double mutant W31FW76F was constructed, and showed a drop off in binding energy from 8.5 kcal.mol^−1^ to 7.1 kcal.mol^−1^. To verify the free energy of binding connected with phenylalanine substitution, a second double mutant (W31AW76F) was prepared and thermodynamic parameters were recalculated using fixed parameters for alanine. The free energy of binding is very close to the one obtained for the W31FW76F mutant (Table 1 and Figure S3). The model for one independent set of sites was preferentially used because the model for two independent sites uses six variables in the fitting procedure, which could lead to meaningless results. One has to notice that ITC experiments must be performed in concentration of protein above K_d_ (optimally 10 to 100 higher) for proper sigmoid curve after integration of heat peaks area. It means that in the case of one-point Ala mutants, the concentration of protein is significantly below a possibility to evaluate the data for the Ala mutated site. Using this approach, some mutants showed slight decrease in enthalpy (ΔH) contribution. The difference on ΔH is around 1 kcal.mol^−1^, which can still be a result of changed conditions during the measurement, as such small differences are quite usual in these experiments.

### Computational part of work

**Figure 2:**
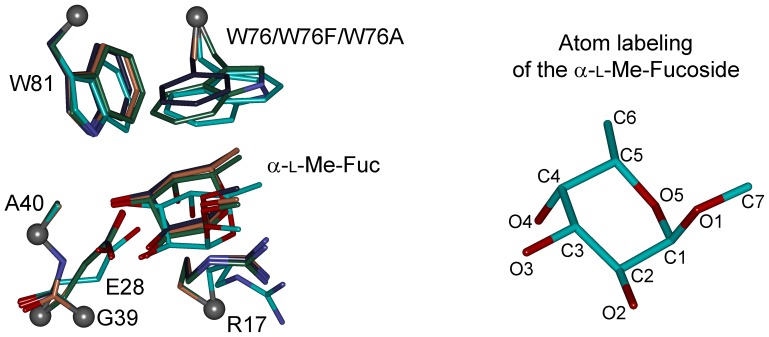
Superimposition of the modeled active site structures. The X-ray structure and the structures of the mutated models' carbons are colored in cyan, optimized BS_W76 model carbons are colored in green, optimized BS_W76F model carbons are colored in violet and optimized BS_W76A model carbons are colored in light brown. Balls represent restrained alpha carbons.

For quantum chemical calculations, binding site models are abbreviated in the text as *BS_W76* (model of the wild type RSL binding site), *BS_W76F* (model of the W76F mutant binding site) and *BS_W76A* (model of the W76A mutant binding site). Optimization of the models led to slight changes in the structure. The largest changes in the structure of the amino acid side chains were observed for the Glu28 and Arg17 residues. These amino acid side chains adopted a slightly different conformation compared to the X-ray structure. Other amino acid residues' side chains did not show any significant changes. The α-l-Me-fucoside residue was slightly shifted deeper into the binding site during the optimization, which led to an increase in favorable contacts with the lectin's amino acid side chains. Superimposition of the optimized models reveals that differences in the geometry of common residues over all modeled structures are negligible (Figure 2). Also, measured distances listed in Table 2 and Table S1 show minimal deviations across all optimized models.

**Table 2:** Measured distances between α-l-Me-fucoside carbon atoms and geometric centers of the aromatic parts of residues for the 76^th^ amino acid.

		Distances [Å]			
76^th^ residue ring center	α-l-Me-Fuc atom	2BT9	*BS_W76*	*BS_W76F* [a]	*BS_W76F*
*W76_Ph_Cent_*/*W76F_Ph_Cent_*	C3	4.151	4.086	5.294	4.279
	C5	3.749	3.334	4.035	3.268
	C6	4.321	3.928	3.806	3.736
*W76_Pyrol_Cent_*	C6	3.700	3.367		

Values in 2BT9 column represent distances in the crystal structure.

[a] Values before geometry optimization.

#### Structural implications of the calculations results

The α-l-Me-fucoside primarily creates two types of intermolecular interactions with RSL. The first is the interaction of the hydrogen bond network with neighboring residues, and the second is the dispersion interaction mainly with tryptophan Trp76. There is also a possible electrostatic interaction between positively charged arginine Arg17 and the α-l-Me-fucoside ring oxygen. Six possible intermolecular hydrogen bonds between the α-l-Me-fucoside and the RSL lectin binding site were identified in the crystal structure. The possibility of hydrogen bonds exists between: the α-l-Me-fucoside's O2 atom and N atom of the Ala40; the O3 atom and OE1 and NE1 atoms of Glu28 and Trp81; the O4 atom and NE and OE2 atoms of Arg17 and Glu28; the O5 atom and NH2 atom of Arg17. All mentioned hydrogen bonds were also retained in the optimized structures of all binding site models. Measured interatomic distances are listed in Table S1. Measured optimized distances are shortened approximately by 0.12 Å compared to the crystal structure, except for the N of Ala40 and the NE1 of Trp81, where distances are slightly elongated. This elongation is caused by the movement of α-l-Me-fucoside deeper into the binding site, which is in a direction away from these residues. Measured interatomic distances range from 2.524 up to 3.199 Å. Hydrogen bonds within this range of distances are thought to be strong and can be found in all modeled binding site optimized structures.

**Figure 3:**
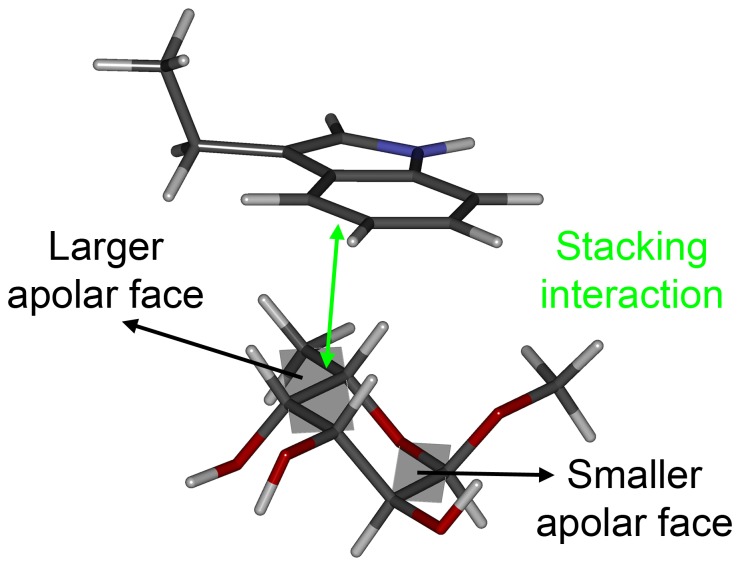
Graphical representation of the α-l-Me-fucoside apolar faces. We can define two apolar faces for the Me-fucoside. The plane defined by C1, C2 and O5 atoms creates smaller one whereas the plane defined by C3, C4, C5 and C6 atoms creates larger one. This larger apolar face creates stacking interaction with Trp76 residue in RSL lectin.

**Figure 4:**
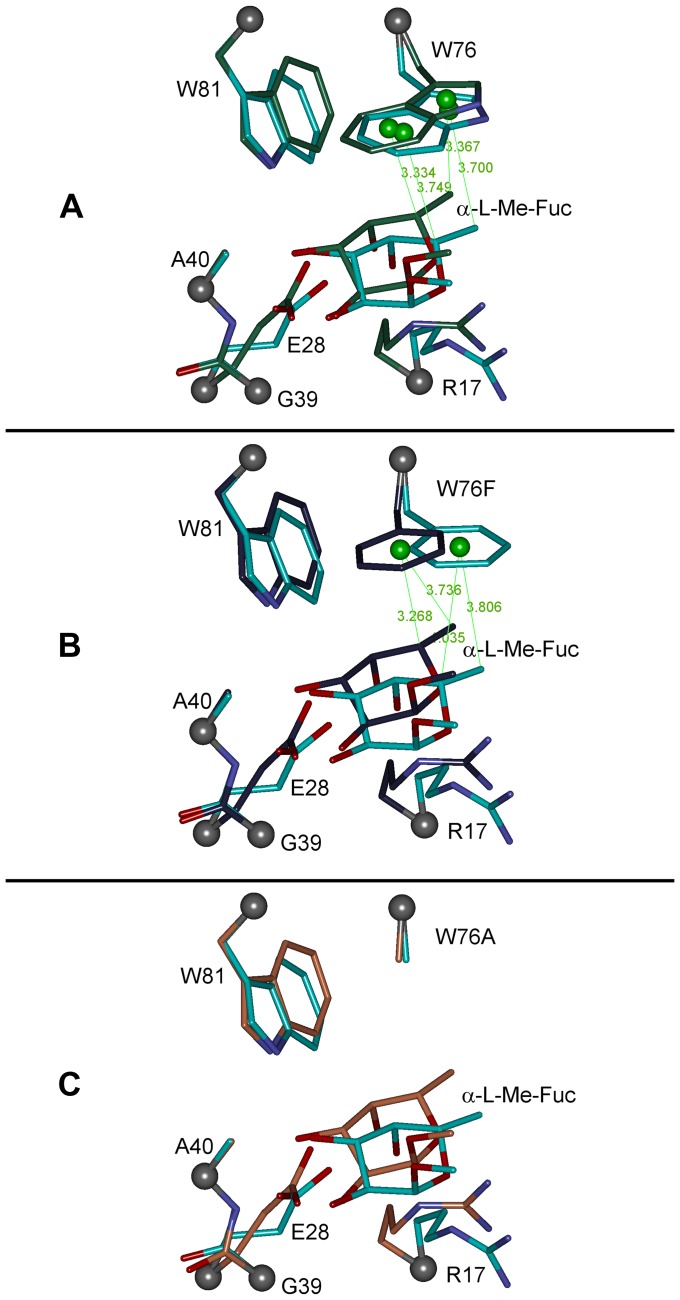
Overlay of the RSL binding site models before and after geometry optimization. Overlay of the wild type binding site model (BS_W76) before (cyan) and after (green) optimization (**A**); RSL W76F mutant binding site model (BS_W76F) before (cyan) and after (violet) optimization (**B**); RSL W76A mutant binding site model (BS_W76A) before (cyan) and after (light brown) optimization (**C**). Selected distances from the geometrical centers of tryptophan's indol part and the phenyl part of phenylalanine geometrical center are shown.

The non-polar face of α-l-Me-fucoside interacts with the aromatic side chain of Trp76 in the crystal structure. The nonpolar face is created by the CH groups on the C3, C4, C5, and C6 carbon atoms (as labeled in Figure 2) of the fucoside ring. The plane defined by these atoms is parallel to the aromatic moiety of Trp76 and the fucoside makes stacking interaction with it (Figure 3). The hydrogen atoms are pointing toward the indole part of the Trp76 residue, except for the CH group on carbon C4, where the hydrogen atom is in equatorial position. The aforementioned CH groups are also pointing towards the phenyl ring of the phenylalanine in the mutated *BS_W76F* model. The point mutant *BS_W76A* model has no aromatic moiety. Measured distances between the C3, C5, C6 carbon atoms and the geometrical center of the present aromatic rings are listed in Table 2. For a better description of possible interactions, the indole part of Trp76 was taken as two aromatic rings with two geometrical centers. One geometrical center is located on the 6-membered ring (named as *W76_Ph_Cent_*) and the second on the pyrrole part (named as *W76_Pyrrole_Cent_*) of the indole moiety. This separation allows for a better comparison of the geometrical parameters of the complex with those of the *BS_W76F* mutant, where only a phenyl ring is present. The distances between C3, C5, C6 and the geometrical center of the 6-membered ring of the Trp76's indole moiety before geometry optimization (*W76_Ph_Cent_*) were 4.151, 3.749 and 4.321 Å, respectively (Table 2, Figure 4A). The distance between the C6 atom and the pyrrole geometrical center (*W76_Pyrrole_Cent_*) was 3.700 Å. After the geometrical optimization of the *BS_W76* model, these distances were shortened to 4.086, 3.334 and 3.928 or 3.367 Å between the C3, C5, C6 and the *W76_Ph_Cent_* or between the C6 and the *W76_Pyrrole_Cent_*, respectively. These distances suggest that the CH groups on the C5 and C6 carbon atoms are strongly involved in the dispersion interaction with the Trp76 residue, where C5 is interacting with the 6-membered ring part and C6 is interacting with the pyrrole part of the Trp76's indole moiety. The *BS_W76F* model's structure was similarly analyzed. The geometrical center of the phenyl moiety in the W76F mutant is named analogously in the *BS_W76* model as *W76F_Ph_Cent_*. Distances between the C3, C5, C6 carbon atoms and *W76F_Ph_Cent_* before optimization were 5.294, 4.035 and 3.806 Å, respectively. Optimization of the *BS_W76F* model leads to the shortening of these distances, in this case to 4.279, 3.268 and 3.736 Å for C3, C5 and C6, respectively (Table 2, Figure 4B). Measured distances in the optimized *BS_W76F* model are comparable to values in the *BS_W76* model for distances with *W76_Ph_Cent_* at the center. The position of the phenyl ring is close to the position of the 6-membered ring of the indole moiety in the optimized *BS_W76* model after the *BS_W76F* model geometry optimization (Figure 2). The distance shortening is caused mainly by the movement of the phenyl ring of the phenylalanine residue. This movement brings the phenyl group to the position where interaction with the C5 and C6 CH groups is more favorable. Moreover, the measured distances indicate that the C5 and C6 CH groups are also preferred in the interaction with phenylalanine. The environment around the C6 methyl group is completed by the Ile59 and Ile61 and by Pro14 and Ile16 in the intra- and intermonomeric sites, respectively. The methyl group makes van der Waals contacts only with these two residues. However, our goal is to calculate the interaction energy between the α-l-Me-fucoside and Trp76. This interaction energy calculation should therefore not be influenced by Ile and Pro amino acid residues. Moreover, their presence in the binding site model could significantly complicate the calculation of the α-l-Me-fucoside - 76^th^ residue interaction energy. These are the reasons why these amino acid residues were excluded from the computational model.

#### Dispersion energy (CH/π) estimation

**Figure 5:**
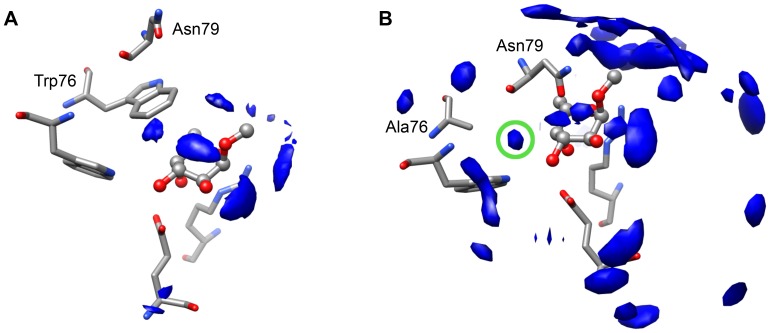
Comparison of the observed water densities from the MD simulation of the wild type RSL and W76A RSL mutant. The water densities in the 34 ns long molecular dynamic simulations of the wild type RSL (**A**) and W76A RSL mutant (**B**) are shown. Detailed analysis of the trajectories show increased water density around the α-L-Me-fucoside moiety in the W76A mutant in position occupied by Trp76 side chain in the wild type lectin complex simulation. This increased water density is highlighted by green circle. The α-L-Me-fucoside is shown in ball and stick representation.

The major interest of the computational study was to calculate the interaction energy between the α-l-Me-fucoside and the Trp76 residue (*BS_W76* model), or its mutated residues phenylalanine (*BS_W76F* model) and alanine (*BS_W76A* model). Interaction energies were calculated as the difference between two monomers. Monomer1 is the 76^th^ residue and monomer2 is a complex constituted by Arg17, Glu28, Gly39, Ala40, Trp81 and α-l-Me-fucoside. However, this methodology does not allow for direct calculations of specific interactions between monomer1 and α-l-Me-fucoside, as the total energy comprises contributions from the other residues in monomer2, most importantly the non-covalent intermolecular interactions with Trp81 through one hydrogen bond. However, Trp81 creates T-shaped CH/π interaction with Trp76. This interaction significantly influences the energy estimation between α-l-Me-fucoside and Trp76 residues (Table S2) in the wild type and also in W76F and W76A mutant models. Therefore, to eliminate the above 76^th^ and Trp81's contact contribution to the interaction energy, we excluded Trp81 from monomer2 and evaluated interaction energy without Trp81. We assume that the main role of the Trp81 residue is to serve as an anchor for the correct position of the Trp76 by the strong T-shaped CH/π interaction and also to create a strong hydrogen bond with the O3 fucoside hydroxyl group, which will always be there, so it will not change the interaction energy of the mutants. Altogether, as we focus on the dispersion interaction of the α-l-Me-fucoside with the Trp76 in our study, the absence of the Trp81 in the model will not influence the estimated interaction energy, and we observed the 76^th^ residue – α-l-Me-fucoside interaction energy only (Table 1). Moreover, the interaction of the 76^th^ residue with other binding site residues does not have any influence on the interaction energy between the 76^th^ residue and α-l-Me-fucoside. Because the interaction energy between α-l-Me-fucoside and Trp76/Phe76 has preferably dispersive character, estimated interaction energy is approximately equal to dispersion (CH/π) energy. In such case, the α-l-Me-fucoside – Trp76/Phe76 interaction energy defines a dispersion part of the total binding energy. Interaction energies obtained with such methodology are discussed below. Calculation of the interaction energy and its comparison to the experimental binding energy clearly shows a strong contribution of the dispersion interaction energy to the overall binding energy between the α-l-Me-fucoside and RSL. Calculated and experimental energies of the α-l-Me-fucoside with the RSL lectine and its mutants are listed in Table 1. Calculated interaction energies for the *BS_W76*, *BS_W76F* and *BS_W76A* models are −8.85, −7.92 and −0.91 kcal.mol^−1^, respectively. Experimentally measured binding energy values are −8.50, −7.04 and −4.14 kcal.mol^−1^ for the wild type RSL, W31FW76F and W31AW76A RSL point mutants, respectively. Comparison of the calculated and experimental values clearly shows that values calculated in the absence of Trp81 very well reproduce the experimental data except for the *BS_W76A* mutant model. To explore the observed inconsistency in our data, we decided to also focus our attention on solvent behavior. Therefore, we ran molecular dynamics simulations of wild type and W76A mutant RSL lectins in an explicit solvent (TIP4P water model) using the AMBER11 program package with parm99sb [48] and GLYCAM06 [49], [50] force fields. Detailed analysis of the 34 ns long production trajectories shows increased water density around the α-l-Me-fucoside moiety in the W76A mutant, in place where the Trp76 side chain is positioned during the wild type lectin complex simulation (Figure 5). During the simulation we also observed Ala76 – Trp81 loop movement above the fucose moiety in one of the active sites of the W76A mutant lectin. This loop movement closes the binding site and brings the side chain of asparagine 79 (Asn79) close to the α-l-Me-fucoside. Then, Asn79 creates a stable hydrogen bond with the O2 hydroxyl for the duration of the simulation (Figure S4 and S5). The difference between the calculated interaction energy and the observed binding energy in the case of the W76A mutant is 3.23 kcal.mol^−1^, which corresponds to a medium-strong hydrogen bond. Molecular dynamics simulations, therefore, show that the observed energy difference for the complex can be caused by a water molecule mediated interaction with lectin or by creation of the hydrogen bond between Asn79 and the α-l-Me-fucoside.

Interpreting the excellent agreement of these calculated energies, in the case of the *BS_W76* and *BS_W76F* models, one may note that the experimental values of the binding energy also contain a solvation/desolvation contribution to the overall energy during the α-l-Me-fucoside binding process, whereas these effects are elided in the calculation. Unfortunately, quantitative observation of the solvation/desolvation energy of the α-l-Me-fucoside is not possible using known computational methodologies. The solvation/desolvation effects in the case of the α-l-Me-fucoside can be significant, due to the large amount of hydrogen bonds the α-l-Me-fucoside may create with the solvent molecules. However, the α-l-Me-fucoside also creates strong hydrogen bonds with RSL when in complex with it. We therefore assume that the binding energy generated by these hydrogen bonds fully compensates for the α-l-Me-fucoside desolvation energy, and the ITC measured binding energy is really a dispersion interaction generated mainly by the interaction of the α-l-Me-fucoside with Trp76. Observed agreement between the calculated dispersion interaction energies and experimental binding energies together with the binding site topology and stoichiometry of the complexes suggest that the solvation/desolvation energy of the fucoside is compensated by the polar and van der Waals interactions with lectin and strong dispersion interaction is the energy benefit which keeps the fucoside strongly in the binding site.

When we assume that polar fraction of the enthalpic part of the binding energy remains the same (i.e., number of hydrogen bonds remains unchanged) then the measured value corresponds to dispersion interaction energy plus entropy change. As the entropy change is around 1 kcal.mol^−1^ (Table 1), the pure dispersion interaction energy is between 7.0 and 8 kcal.mol^−1^.

## Conclusion

In the study presented here, we have attempted to quantify the contribution of the dispersion CH/π interaction to the binding of the α-l-Me-fucoside to the RSL lectin. The CH/π interaction is thought to be held between the fucose apolar plane and Trp31 or Trp76 in the binding site. The single and double point mutants of Trp residues clearly show a significant decrease in the binding affinity. In the case of W31FW76F, a decrease in binding energy, from −8.5 to −7.1 kcal.mol^−1^, is not as high as in the case of the double alanine mutant W31AW76A (from −8.5 to −4.1 kcal.mol^−1^), because some CH/π interactions between the fucoside moiety and phenylalanine are still present, as shown by the optimization of the *BS_W76F* binding site model. The largest difference between measured and calculated interaction energies was observed for the alanine mutant, where the measured binding energy was −4.1 and the calculated interaction energy was only −0.9 kcal.mol^−1^. In this case, the molecular dynamics simulation shows that the observed difference can be caused by increased water density in the place of mutated tryptophan, or by movement of a close loop and creation of a new hydrogen bond with the asparagine residue. Both scenarios are possible and correspond well to the energy difference between measured and calculated energies. That difference corresponds to the energy of a medium-strong hydrogen bond. ITC measured entropy contribution is 1 kcal.mol^−1^. In the assumption that polar part of the enthalpy did not change after the binding, we can conclude that contribution of the dispersion interaction to the binding is between 7.0 and 8.0 kcal.mol^−1^. Observed results suggest that, in the case of the RSL lectin, interaction with the α-l-Me-fucoside is strongly driven by the dispersion interaction. In our opinion, such a conclusion might be further generalized to describe similar carbohydrate binding sites where a strong dispersion interaction can occur between a carbohydrate and an aromatic amino acid residue. The obtained results also suggest that polar interactions of sugar hydroxyl groups in the receptor protein serve to counterbalance the carbohydrate desolvation effect.

## Experimental Section

### Computational details

The crystal structure of the fucose binding lectin from *Ralstonia solanacearum* (RSL lectin; PDB ID: 2BT9) [42] served as a template for all of our binding site models. The structure contains three monomer units of the lectin with six binding sites, where three of them are intramonomeric and the other three are intermonomeric. These binding sites differ only in one amino acid residue, where Ile59 in the intramonomeric binding site is replaced by Pro14 from the neighboring protein chain in the intermonomeric binding site (Figure S1). The structure of the intramonomeric and intermonomeric binding sites is almost identical. Therefore, we do not expect that the intermonomeric binding site will behave differently during the geometry optimization and the intramonomeric binding site was chosen for the modeling study. A binding site model containing the α-l-Me-fucoside, Arg17, Glu28, Gly39, Ala40, Trp76 and Trp81 residues was used in the calculations (abbreviated in the text as *BS_W76*). Because the Pro14/Ile59 residues make hydrophobic contact with the methyl group of the fucose moiety, they were excluded from the binding site models. Their absence in the models has no influence on the CH/π interaction energy estimation. Models of two point mutants, Trp76Phe and Trp76Ala, were also created. The models of these mutants were prepared *in silico* by manual replacement of Trp76 by the Phe (abbreviated in the text as *BS_W76F*) or Ala (abbreviated in the text as *BS_W76A*) amino acid residues. Both residues were placed in the position where their common atoms with the Trp76 residue had the same positions. The geometry structure of all prepared RSL binding site models was optimized. The alpha carbons of all amino acid residues were fixed to their crystallographic positions during the optimization, and the rest of the model was fully optimized without any restraints or constraints. The geometry optimization was done employing the Density Functional Theory with Grimmes's empirical corrections [51], [52] to the dispersion energy (DFT-D). The Becke-Perdew functional [53]–[55] with triple-ζ quality basis set def2-TZVPP implemented in the TURBOMOLE program package was used. All calculations were performed in the TURBOMOLE 6.0 program package [56], [57] employing the resolution of identity for DFT calculation algorithm [58]–[60] (ri-dft routine in TURBOMOLE package). The interaction energies for all optimized models were calculated with the basis set superposition error correction [61], [62] as is implemented in the TURBOMOLE program at the same level of theory.

### Experimental details

#### Mutagenesis of RSL in positions 31 and/or 76

A plasmid construct named *pET25rsl*, containing the plasmid pET-25(b+) (Novagen) and the full-length wild type *R. solanacearum* RSL encoding gene [42], was used as the initial template for single site mutagenesis. Site-directed mutagenesis was performed with the QuickChangeTM Site-Directed Mutagenesis Kit (Stratagene), following the manufacturer's instructions. Double mutants were constructed using plasmids of corresponding single mutants. The oligonucleotides and templates used for the mutations are summarized in the Table S3. The new constructs were transformed into the *E. coli* Tuner(DE3) strain (Novagen). The wild type RSL lectin and all mutants were expressed in E. coli Tuner(DE3) cells and purified on a mannose-agarose column as previously described [42]. The purified proteins were stored at −20°C in a lyophilized form.

#### Microcalorimetry

Isothermal titration calorimetry (ITC) experiments were performed using ITC_200_ microcalorimeter (GE Healthcare). All titrations were performed in 0.1 M Tris/HCl buffer, pH 7.5 at 25°C. Aliquots of 2 µl of α-l-Me-Fuc (1 mM) dissolved in the same buffer were added at 4 min intervals to the lectin solution (0.06 mM) present in the calorimeter cell. At least three independent titrations were performed for each ligand tested except for the double mutant W31AW76A, where final yields of the purified proteins were too low. The temperature of the cell was controlled to 25±0.1°C. Control experiments performed by injections of buffer in the protein solution yielded to insignificant heats of dilution. Integrated heat effects were analysed by non-linear regression using a single site-binding model (Microcal Origin 7). Fitted data yielded the association constant (*K*_a_) and the enthalpy of binding (Δ*H*). Other thermodynamic parameters, i.e. changes in free energy, Δ*G*, and entropy, Δ*S*, were calculated from Equation (1),
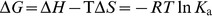
(1)where *T* is the absolute temperature and *R* = 8.314 J.mol^−1^.K^−1^.

#### Size-exclusion chromatography

Possible changes in oligomeric state of the proteins after mutagenesis were checked by size-exclusion chromatography, using a Superose 12 column (GE Healthcare) in 20 mM Tris/HCl and 0.3 mM NaCl (pH 7.5) and a flow rate of 0.75 ml/min. A calibration curve for molecular size estimation was generated from elution volumes of individually loaded cytochrome c, myoglobin, ovalbumin and BSA.

#### Analytical ultracentrifugation

Sedimentation analyses of RSL and RSL W31A were performed using a ProteomeLab XL-A analytical ultracentrifuge (Beckman Coulter) equipped with an An-60 Ti rotor. Before analysis, lyophilized proteins were dissolved in the same experimental buffer (20 mM Tris/HCl, 150 mM NaCl, pH 7.3) that was used as a reference. Sedimentation velocity experiments were conducted in a standard double-sector centerpiece cell loaded with 360 µl of protein sample and 380 µl of reference solution. Data were collected using absorbance optics at 25°C and a rotor speed of 40,000 rpm. Scans were performed at 280 nm, 8 min intervals and 0.003 cm spatial resolution in continuous scan mode. The partial specific volumes of proteins, together with solvent densities and viscosities, were calculated from amino acid sequences and buffer composition, respectively, using the software Sednterp 1.09 (www.rasmb.bbri.org). The sedimentation profiles were analyzed with the program Sedfit 12.1 [63]. A continuous size-distribution model for non-interacting discrete species providing a distribution of apparent sedimentation coefficients was used.

## Supporting Information

Figure S1 **Overlay of the intramonomeric and intermonomeric RSL lectin binding sites created from 2BT9 pdb structure.** Residues in the intramonomeric binding site are colored with gray carbon atoms and gray labels. Residues in the intermonomeric binding site are colored with cyan carbons and cyan labels. Overlay was created by superimposition of the α-l-Me-fucoside atoms.
(TIF)

Figure S2 **Determination of oligomeric state of wild type RSL and W31A mutant by analytical ultracentrifuge.** Sedimentation profiles and the fitted curves of RSL (0.16 mg.ml^−1^) (**A**) and RSL W31A (0.17 mg.ml^−1^) (**B**) obtained from continuous c(s) analysis using Sedfit are shown in upper panel. Sedimentation velocity experiments were carried out at 40,000 rpm at 25°C and the scans were recorded every 8 minutes. For simplicity every third scan is shown, the last profile corresponds to 5 hours of sedimentation. Residual plot (middle panel) shows the differences between the experimental and fitted curves. Continuous size-distribution of sedimenting species (lower panel) provided a value of sedimentation coefficient of 3.23±0.02 S for RSL and 3.19±0.02 S for RSL W31A. Data analysis of RSL measurement provided a single peak corresponding to 3.23±0.02 S (s^0^_20,w_ = 3.00 as calculated using Sednterp). The value is clearly much higher than the predicted maximum value for spherical monomer (1.76 S) or dimer (2.80 S, as calculated in Sednterp) suggesting that a trimer is formed. The result is consistent with the value of sedimentation coefficient of 3.19±0.02 S (s^0^_20,w_ = 2.96 S) obtained for RSL W31A and gives an evidence that mutation W31A does not affect the protein oligomeric state. The frictional coefficient ratios f/f_0_ for RSL and RSL W31A were calculated to be 1.21 and 1.22, respectively, that are common values for globular, hydrated proteins.
(TIF)

Figure S3 **Microcalorimetry data.** ITC plot (measured by ITC_200_, GE Healthcare) obtained for the double mutant W31AW76F (0.06 mM) titrated by 2 µl aliquots of α-L-Me-fucoside (1 mM) at 25°C. The lower plots show the total heat released as a function of total ligand concentration for the titration shown in panel up. The solid line represents the best least-square fit to experimental data using one site (left) and two sites (right) models, respectively. The calculated thermodynamic parameters for both models are shown in the Table 1.
(TIF)

Figure S4 **Visualization of the hydrogen bond between Asn79 and O2 hydroxyl group of the α-l-Me-fucoside created during the W76A mutated RSL lectin molecular dynamic simulation.**
(TIF)

Figure S5 **Plot of distance between the OD1 atom of the Asn79 and O2 oxygen during the W76A mutated RSL lectin molecular dynamic simulation.**
(TIF)

Table S1 **Measured optimized hydrogen bond distances between the α-l-Me-fucoside and RSL binding site amino acid residues for all model structures.** Values in 2BT9 column represent distances in the crystal structure.
(DOC)

Table S2 **Comparison of the calculated interaction energies (** ***E*** **_Int_) between monomer1 and monomer2 with or without presence of the Trp81.** The experimental binding energies (*E*_Int-Exp_) are also listed.
(DOC)

Table S3 **Primers used for RSL mutagenesis.** Nucleotide substitution triad is in bold. First, all single-point mutants were constructed. Then, mutants in position 76 served as a templates for the second mutagenesis using appropriate primers to create double-mutants.
(DOC)
