# Supplementary figures and images for: Stacking Interactions between Carbohydrate and Protein Quantified by Combination of Theoretical and Experimental Methods
Source: PLoS One. 2012 Oct 8;7(10):e46032. doi: 10.1371/journal.pone.0046032 (PMC3466270; doi:10.1371/journal.pone.0046032)

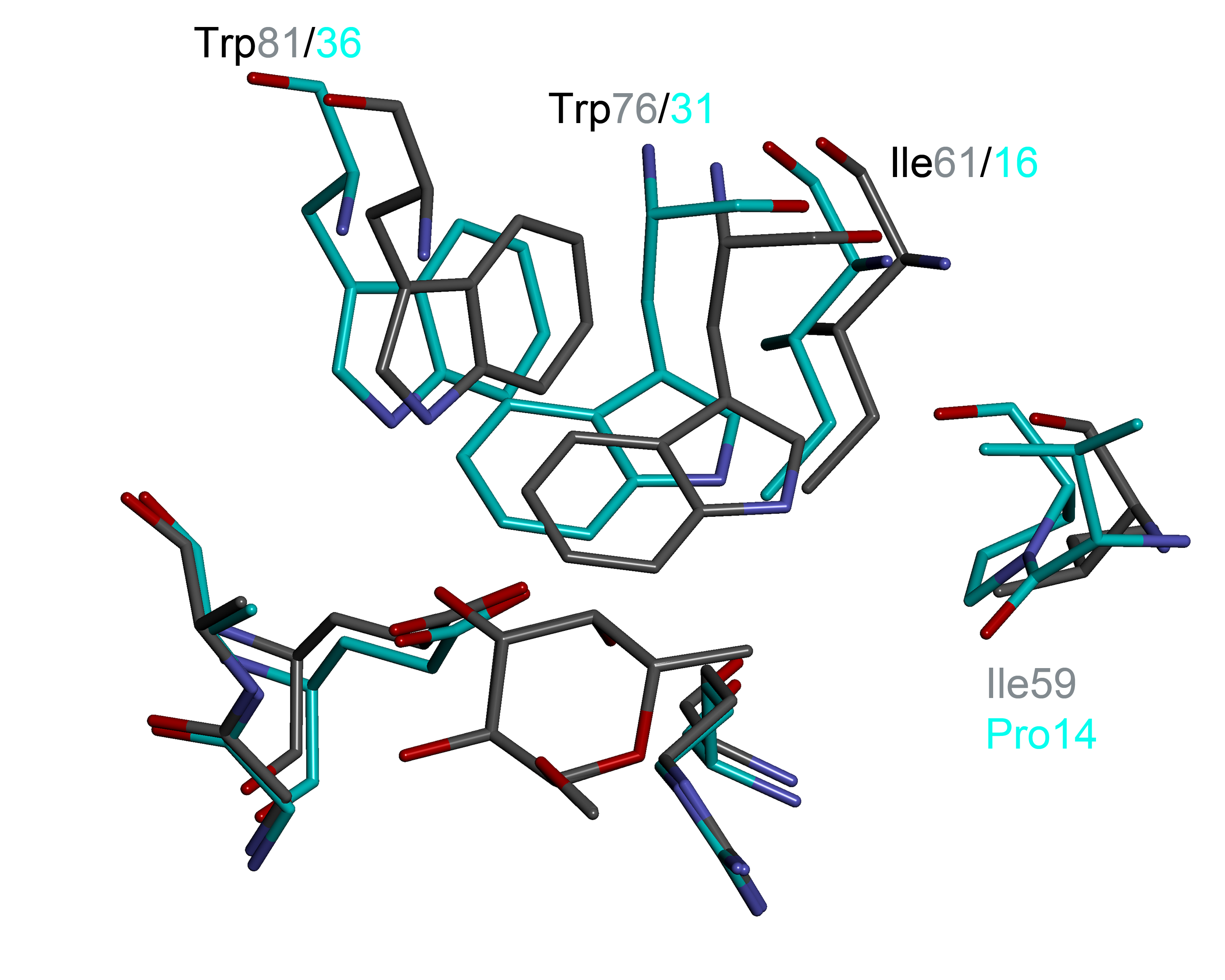

Supplement: Figure S1 — Overlay of the intramonomeric and intermonomeric RSL lectin binding sites created from 2BT9 pdb structure. Residues in the intramonomeric binding site are colored with gray carbon atoms and gray labels. Residues in the intermonomeric binding site are colored with cyan carbons and cyan labels. Overlay was created by superimposition of the α-l-Me-fucoside atoms. (TIF) [file pone.0046032.s001.tif]

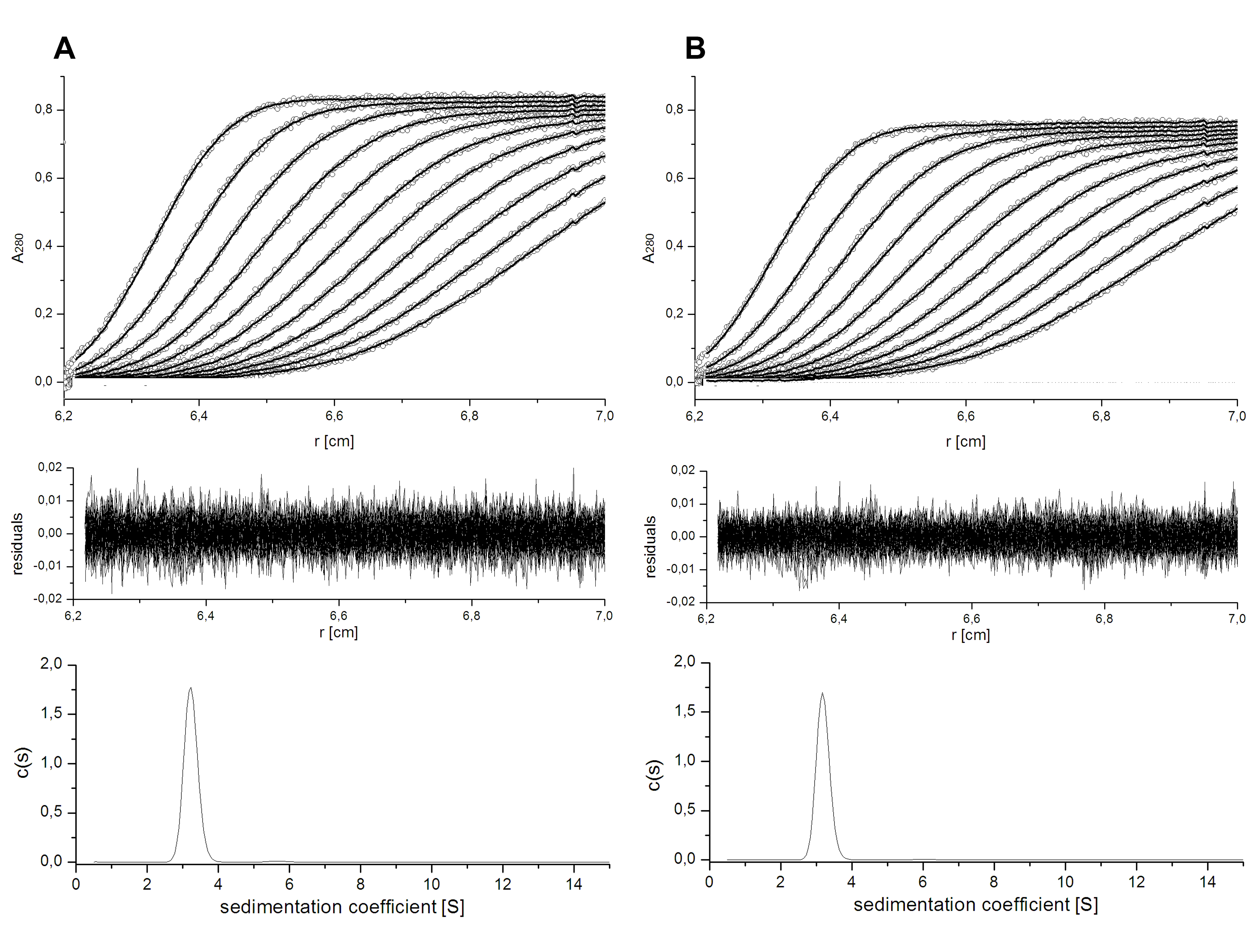

Supplement: Figure S2 — Determination of oligomeric state of wild type RSL and W31A mutant by analytical ultracentrifuge. Sedimentation profiles and the fitted curves of RSL (0.16 mg.ml−1) (A) and RSL W31A (0.17 mg.ml−1) (B) obtained from continuous c(s) analysis using Sedfit are shown in upper panel. Sedimentation velocity experiments were carried out at 40,000 rpm at 25°C and the scans were recorded every 8 minutes. For simplicity every third scan is shown, the last profile corresponds to 5 hours of sedimentation. Residual plot (middle panel) shows the differences between the experimental and fitted curves. Continuous size-distribution of sedimenting species (lower panel) provided a value of sedimentation coefficient of 3.23±0.02 S for RSL and 3.19±0.02 S for RSL W31A. Data analysis of RSL measurement provided a single peak corresponding to 3.23±0.02 S (s0 20,w = 3.00 as calculated using Sednterp). The value is clearly much higher than the predicted maximum value for spherical monomer (1.76 S) or dimer (2.80 S, as calculated in Sednterp) suggesting that a trimer is formed. The result is consistent with the value of sedimentation coefficient of 3.19±0.02 S (s0 20,w = 2.96 S) obtained for RSL W31A and gives an evidence that mutation W31A does not affect the protein oligomeric state. The frictional coefficient ratios f/f0 for RSL and RSL W31A were calculated to be 1.21 and 1.22, respectively, that are common values for globular, hydrated proteins. (TIF) [file pone.0046032.s002.tif]

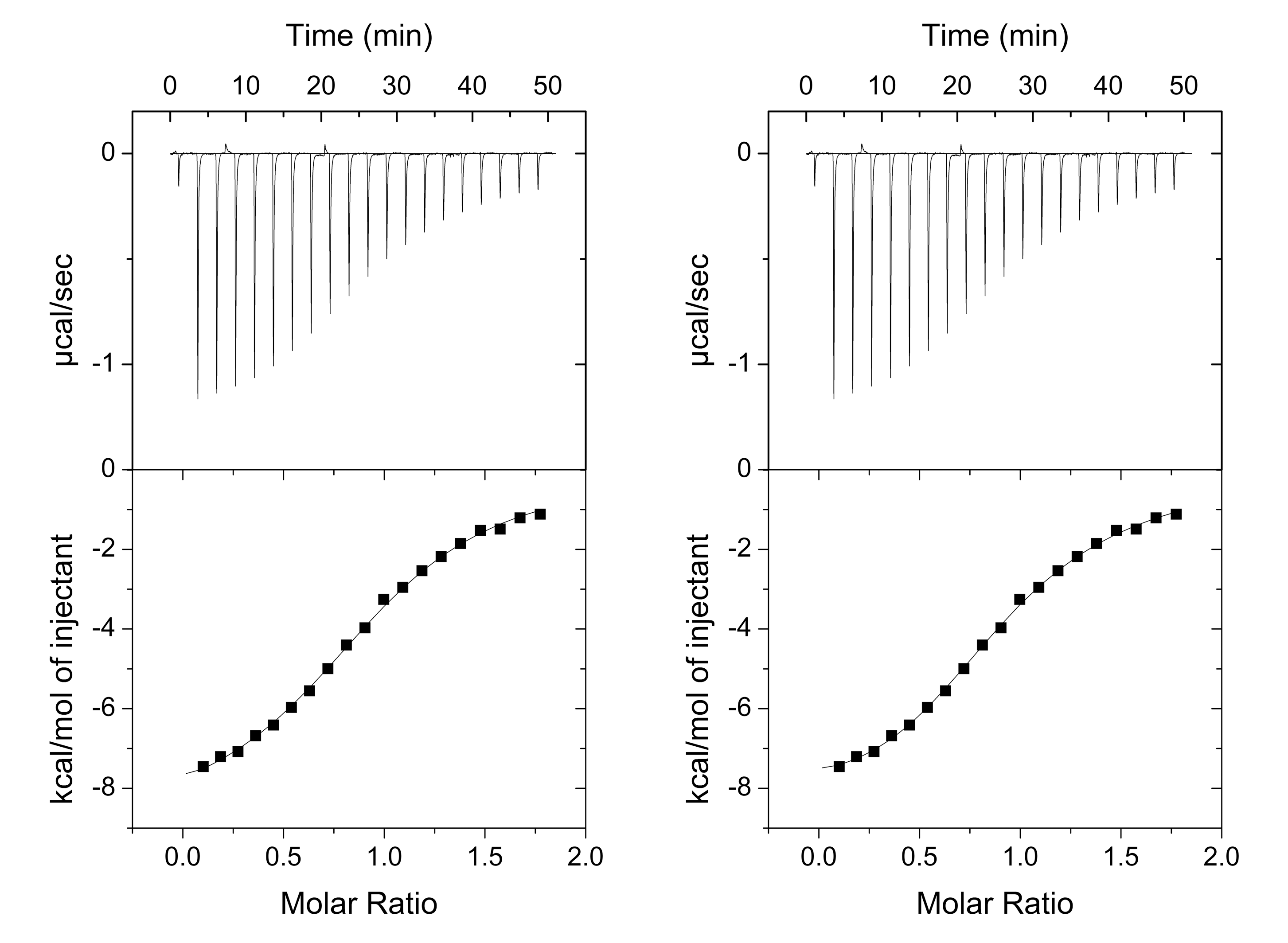

Supplement: Figure S3 — Microcalorimetry data. ITC plot (measured by ITC200, GE Healthcare) obtained for the double mutant W31AW76F (0.06 mM) titrated by 2 µl aliquots of α-L-Me-fucoside (1 mM) at 25°C. The lower plots show the total heat released as a function of total ligand concentration for the titration shown in panel up. The solid line represents the best least-square fit to experimental data using one site (left) and two sites (right) models, respectively. The calculated thermodynamic parameters for both models are shown in the Table 1. (TIF) [file pone.0046032.s003.tif]

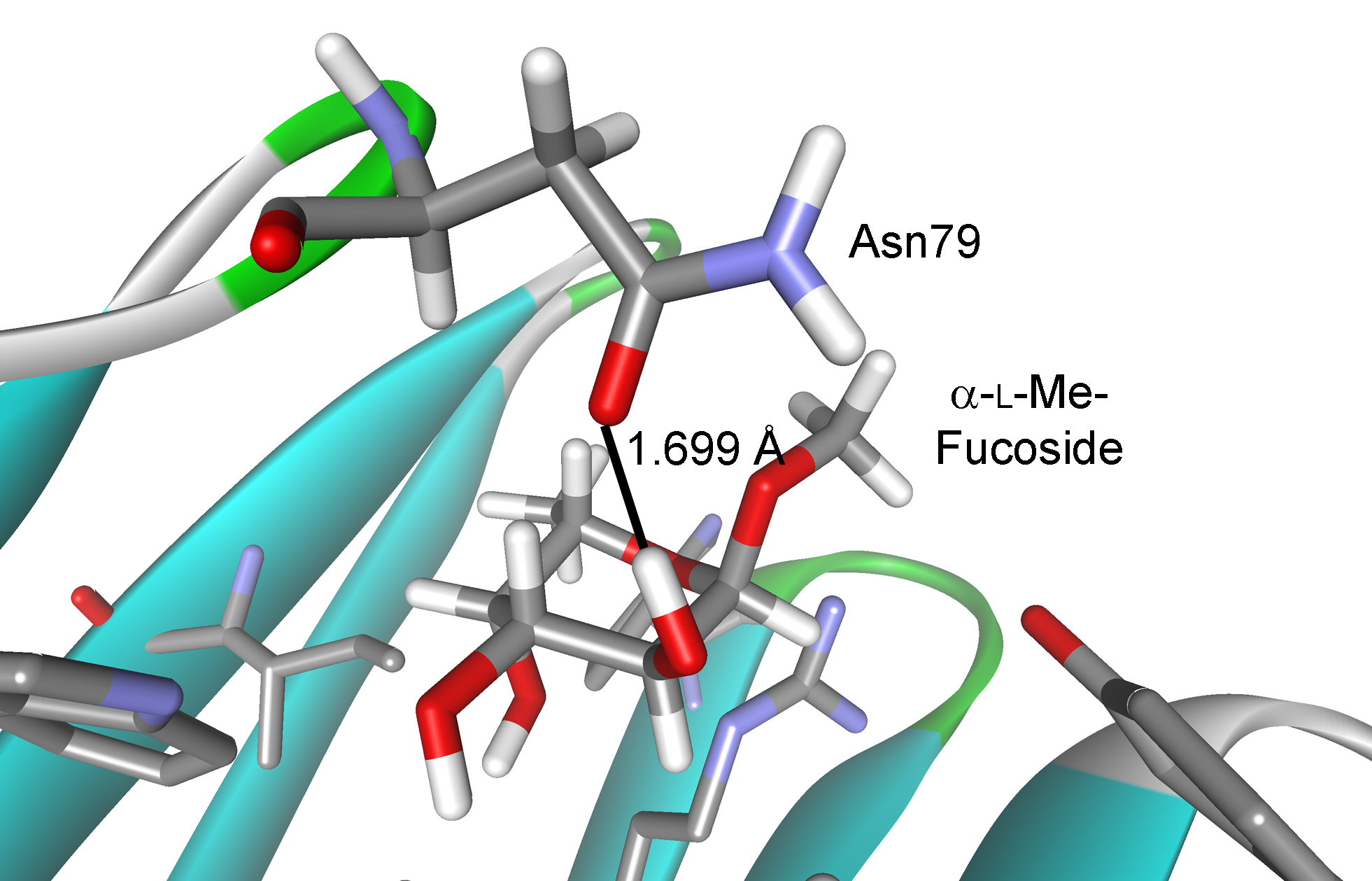

Supplement: Figure S4 — Visualization of the hydrogen bond between Asn79 and O2 hydroxyl group of the α-l-Me-fucoside created during the W76A mutated RSL lectin molecular dynamic simulation. (TIF) [file pone.0046032.s004.tif]

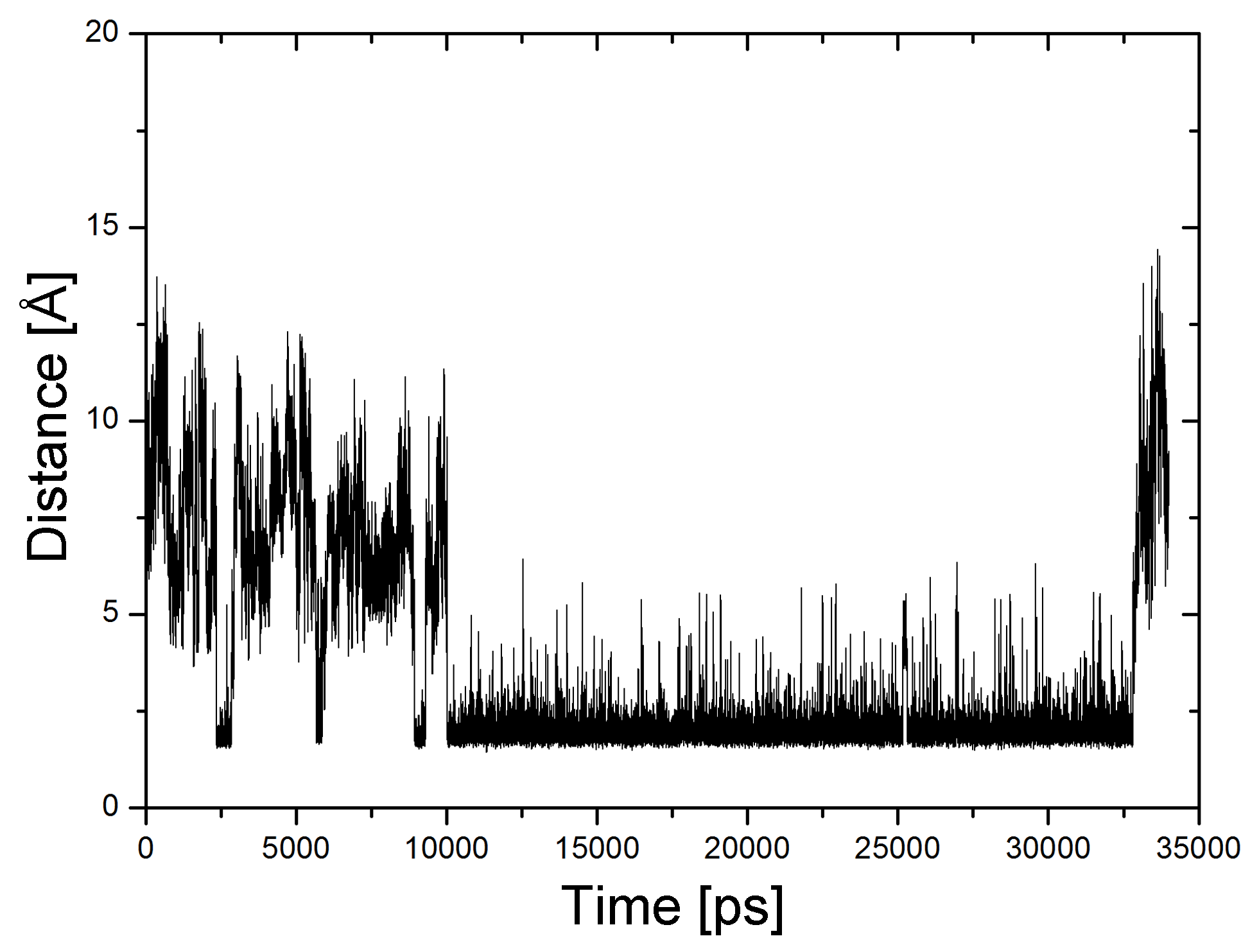

Supplement: Figure S5 — Plot of distance between the OD1 atom of the Asn79 and O2 oxygen during the W76A mutated RSL lectin molecular dynamic simulation. (TIF) [file pone.0046032.s005.tif]
