# Supplementary material for: Stacking Interactions between Carbohydrate and Protein Quantified by Combination of Theoretical and Experimental Methods
Source: PLoS One. 2012 Oct 8;7(10):e46032. doi: 10.1371/journal.pone.0046032 (PMC3466270; doi:10.1371/journal.pone.0046032)
Supplement: Table S1 — Measured optimized hydrogen bond distances between the α-l-Me-fucoside and RSL binding site amino acid residues for all model structures. Values in 2BT9 column represent distances in the crystal structure. (DOC) [file pone.0046032.s006.doc]

|  |  |  | Distances [Å] | | | |
| --- | --- | --- | --- | --- | --- | --- |
| Residue | Atom | α-l-Me-Fuc atom | 2BT9 | *BS_W76* | *BS_W76F* | *BS_W76A* |
| R17 | NE | O4 | 2.854 | 2.639 | 2.633 | 2.645 |
|  | NH2 | O5 | 2.906 | 2.747 | 2.748 | 2.756 |
| E28 | OE1 | O3 | 2.608 | 2.583 | 2.576 | 2.556 |
|  | OE2 | O4 | 2.641 | 2.533 | 2.524 | 2.538 |
| A40 | N | O2 | 2.901 | 3.063 | 3.147 | 3.199 |
| W81 | NE1 | O3 | 2.792 | 2.812 | 2.788 | 2.836 |
