# Supplementary material for: Stacking Interactions between Carbohydrate and Protein Quantified by Combination of Theoretical and Experimental Methods
Source: PLoS One. 2012 Oct 8;7(10):e46032. doi: 10.1371/journal.pone.0046032 (PMC3466270; doi:10.1371/journal.pone.0046032)
Supplement: Table S2 — Comparison of the calculated interaction energies ( E Int) between monomer1 and monomer2 with or without presence of the Trp81. The experimental binding energies (E Int-Exp) are also listed. (DOC) [file pone.0046032.s007.doc]

|  |  | *E*_Int_ [kcal/mol] | *E*_Int-Exp_ [kcal/mol] |
| --- | --- | --- | --- |
| W81 present | *BS_W76* | -17.00 | -8.50 |
|  | *BS_W76F* | -16.40 | -7.04 |
|  | *BS_W76A* | -4.15 | -4.14 |
| W81 absent | *BS_W76* | -8.85 | -8.50 |
|  | *BS_W76F* | -7.92 | -7.04 |
|  | *BS_W76A* | -0.91 | -4.14 |
