# Supplementary material for: Stacking Interactions between Carbohydrate and Protein Quantified by Combination of Theoretical and Experimental Methods
Source: PLoS One. 2012 Oct 8;7(10):e46032. doi: 10.1371/journal.pone.0046032 (PMC3466270; doi:10.1371/journal.pone.0046032)
Supplement: Table S3 — Primers used for RSL mutagenesis. Nucleotide substitution triad is in bold. First, all single-point mutants were constructed. Then, mutants in position 76 served as a templates for the second mutagenesis using appropriate primers to create double-mutants. (DOC) [file pone.0046032.s008.doc]

| Primer name | sequence |
| --- | --- |
| W31A | caccgagcgatgc**gcg**gacgggaagggg |
| W31A_anti | ccccttcccgtc**cgc**gcatcgctcggtg |
| W31F | caccgagcgatgc**ttc**gacgggaaggggtg |
| W31F_anti | caccccttcccgtc**gaa**gcatcgctcggtg |
| W76A | cacggagtggtgc**gcg**gacggcaacggc |
| W76A_anti | gccgttgccgtc**cgc**gcaccactccgtg |
| W76F | cacggagtggtgc**ttc**gacggcaacggctg |
| W76F_anti | cagccgttgccgtc**gaa**gcaccactccgtg |
